# Acupuncture for postoperative gastrointestinal dysfunction in cancer: a systematic review and meta-analysis

**DOI:** 10.3389/fonc.2023.1184228

**Published:** 2023-06-09

**Authors:** Dezhi Lin, Yangxu Ou, Longlong Li, Kexin Wu, Qiang Zhang, Jiayin Yan, Kunlin Kuang, Dezhong Peng

**Affiliations:** School of Acupuncture and Tuina, Chengdu University of Traditional Chinese Medicine, Chengdu, China

**Keywords:** acupuncture, postoperative gastrointestinal dysfunction, cancer, systematic review, meta-analysis

## Abstract

**Background:**

Postoperative gastrointestinal dysfunction (PGD) in cancer is the commonest and most severe postoperative complication in patients with cancer. Acupuncture has been widely used for PGD in cancer. This study aimed to evaluate the efficacy and safety of acupuncture for PGD in cancer.

**Methods:**

We comprehensively searched eight randomised controlled trials (RCTs) of acupuncture for PGD in cancer published until November 2022. Time to first flatus (TFF) and time to first defecation (TFD) were the primary outcomes, and time to bowel sound recovery (TBSR) and the length of hospital stay (LOS) were the secondary outcomes. The Cochrane Collaboration Risk of Bias Tool was used to assess the quality of the RCTs, and the Grading of Recommendations Assessment, Development, and Evaluations (GRADE) system was used to evaluate the certainty of the evidence. The meta-analysis was performed using RevMan 5.4, and a publication bias test was performed using Stata 15.1.

**Results:**

Sixteen RCTs involving 877 participants were included in this study. The meta-analysis indicated that acupuncture could effectively reduce the TFF, TFD, and TBSR compared with routine treatment (RT), sham acupuncture, and enhanced recovery after surgery (ERAS). However, acupuncture did not shorten the LOS compared with RT and ERAS. The subgroup analysis revealed that acupuncture could significantly reduce the TFF and TFD. Acupuncture effectively reduced the TFF and TFD in all cancer types included in this review. Besides, local acupoints in combination with distal acupoints could reduce the TFF and TFD, and distal–proximal acupoints could significantly reduce the TFD. No trial reported adverse events of acupuncture.

**Conclusions:**

Acupuncture is an effective and relatively safe modality for treating PGD in cancer. We anticipate that there will be more high-quality RCTs involving more acupuncture techniques and cancer types, focusing on combining acupoints for PGD in cancer, further determining the effectiveness and safety of acupuncture for PGD in patients with cancer outside China.

**Systematic review registration:**

https://www.crd.york.ac.uk/prospero, identifier CRD42022371219.

## 1 Introduction

Cancer is the leading cause of death and a significant barrier to increasing life expectancy worldwide (1). According to the recent data published by the International Agency for Research on Cancer, Lyon, France, in 2021, the number of new cancer cases worldwide reached nearly 20 million in 2020, and the number of new cases worldwide is expected to reach 28.4 million in 2040 (2).

Currently, the main treatment modalities for cancer are surgery, chemotherapy, and radiotherapy. Although the curative effect is rapid, some drawbacks exist. The goal of cancer treatment is not only to eliminate the tumour lesions but also to improve the patient’s quality of life during treatment, which has become a matter of global concern (3). Postoperative gastrointestinal dysfunction (PGD) is one of the main complications after cancer surgery (4, 5). PGD refers to the temporary suspension of gastrointestinal peristaltic coordination after surgical intervention, preventing the effective transport of intestinal contents or tolerance *via* oral feeding (6). The main clinical manifestations of PGD are abdominal distension, abdominal pain, nausea and vomiting, the disappearance of bowel sounds, no exhaust and defecation, and inability to eat orally (6), thereby increasing the risk of postoperative complications, such as intestinal adhesion and intestinal obstruction. This affects postoperative recovery, reduces the patient’s quality of life, and imposes a serious economic and medical resource burden. The gold standard for evaluating the recovery of gastrointestinal function of patients postoperatively is the time to first flatus (TFF) and time to first defecation (TFD) (7, 8).

Acupuncture is a unique, traditional treatment modality in China. Due to its non-pharmacological and minimally invasive characteristics, acupuncture is widely applied for treating various gastrointestinal diseases, including irritable bowel syndrome (9, 10), gastroparesis (11, 12), and constipation (13). Studies have reported the significant efficacy of acupuncture for treating PGD (14). The underlying mechanism for this might be the regulation of the abnormal brain and intestinal peptide levels, thereby promoting postoperative gastrointestinal functional recovery (15). However, these studies focus on acupuncture for PGD in a single cancer type, such as gastric cancer or colorectal cancer. There is no systematic review and meta-analysis that included all cancer types. Therefore, evidence on acupuncture for PGD in cancer is scarce.

Therefore, to provide evidence supporting the clinical recommendation of acupuncture for PGD in cancer, this systematic review and meta-analysis focused on patients with PGD in cancer to critically evaluate the efficacy and safety of acupuncture for PGD in cancer.

## 2 Methods

### 2.1 Registration

This systematic review was performed in accordance with the Cochrane Handbook for Systematic Reviews of Interventions (16) and followed the Preferred Reporting Items for Systematic Reviews and Meta-Analyses guidelines (17). The registered study protocol of this systematic review and meta-analysis was published in PROSPERO (registration number: CRD42022371219).

### 2.2 Search strategy

Two authors (YO and KW) independently performed a systematic literature search of the following databases: PubMed, Cochrane Library, EMBASE, Web of Science, China National Knowledge Infrastructure, Chongqing VIP Database, WanFang Database, and Chinese Biomedical Literature Database. The search strategy comprised three components, which were as follows: 1) clinical condition ([neoplasms OR cancer OR tutor OR malignancy] AND [postoperative complications OR postoperative] AND [gastrointestinal diseases OR gastrointestinal dysfunction]); 2) intervention (acupuncture therapy OR acupuncture OR moxibustion); 3) study type (randomised controlled trial [RCT]). The databases were searched from inception to 4 November 2022. The PubMed search strategy is presented in **Table 1**; this strategy was modified appropriately for other databases. The authors also searched the reference lists of relevant primary and review articles to identify cited articles not detected in electronic searches.

**Table 1:** Search strategy for the PubMed database.

#1	“Acupuncture Therapy”[MeSH Terms]
#2	(((((((((((((((((Embedding[Title/Abstract])) OR (Intradermal Needle[Title/Abstract])) OR (Electroacupuncture[Title/Abstract])) OR (Specific region Acupunture[Title/Abstract])) OR (Eye Acupunture[Title/Abstract])) OR (Scalp Acupunture[Title/Abstract])) OR (Auricular Acupunture[Title/Abstract])) OR (Wrist-ankle Acupunture[Title/Abstract])) OR (Moxibustion[Title/Abstract])) OR (Needle warming therapy[Title/Abstract])) OR (Electric stimulation therapy[Title/Abstract])) OR (Transcutaneous Electric Nerve Stimulation[Title/Abstract])) OR (Transcranial Direct Current Stimulation[Title/Abstract]))
#3	#1 OR #2
#4	“Neoplasms”[MeSH Terms]
#5	((((((((((((Neoplasm[Title/Abstract])) OR (cancer[Title/Abstract])) OR (cancers[Title/Abstract])) OR (Neoplasia[Title/Abstract])) OR (Neoplasias[Title/Abstract])) OR (Malignant Neoplasm[Title/Abstract])) OR (Malignancy[Title/Abstract])) OR (Malignancies[Title/Abstract])) OR (Malignant Neoplasms[Title/Abstract])) OR (Benign Neoplasms[Title/Abstract])) OR (Benign Neoplasm[Title/Abstract])))
#6	#4 OR #5
#7	“Postoperative Complications”[MeSH Terms]
#8	(((((Postoperative[Title/Abstract])) OR (Post-operative[Title/Abstract])) OR (Post-surgical[Title/Abstract])))
#9	#7 OR #8
#10	“Gastrointestinal Diseases”[MeSH Terms]
#11	(((((Gastrointestinal Disease[Title/Abstract])) OR (Gastrointestinal Disorders[Title/Abstract])) OR (Gastrointestinal Disorder[Title/Abstract])) OR (Gastrointestinal Dysfunction[Title/Abstract])))
#12	#10 OR #11
#13	“Randomized Controlled Trial”[MeSH Terms]
#14	((((Clinical Study[Title/Abstract])) OR (Clinical Trial[Title/Abstract])) OR (Controlled Clinical Trial[Title/Abstract]))
#15	#13 OR #14
#16	#3 AND #6 AND #9 AND #12 AND #15

### 2.3 Study selection

#### 2.3.1 Eligibility criteria

1) Patients with PGD in cancer (any cancer type) (patient aged >18 years).
2) Intervention(s): acupuncture therapies, including manual acupuncture (MA), electroacupuncture (EA), body acupuncture, warm needle acupuncture, auricular acupuncture, scalp acupuncture, elongated needle acupuncture, intradermal needle acupuncture, and moxibustion, or acupuncture therapies combined with routine treatment (RT). RT included usual postoperative care, fasting, gastrointestinal decompression, electrolyte acid-base imbalance correction, nutritional support, anti-infection, etc.
3) Comparisons: RT, sham acupuncture (SA), or other therapies.
4) Outcomes: primary outcomes were TFF and TFD, and secondary outcomes were time to bowel sound recovery (TBSR) and the length of hospital stay (LOS).
5) RCTs only.

#### 2.3.2 Exclusion criteria

1) Studies with repeated data or secondary analysis.
2) Studies from non-RCTs (including animal studies, master’s and doctoral dissertations, books, protocols, conference abstracts, case reports, correspondences, overviews, or systematic reviews).
3) Non-cancer PGD.
4) The therapy of the intervention group was not acupuncture or moxibustion or acupuncture combined with other therapies.
5) The intervention group received traditional Chinese medicine (TCM) and ready-for-use TCM.
6) Outcome indicators did not match.

### 2.4 Data extraction

Two investigators (LL and QZ) independently extracted data from the included studies. The extracted data included the reference identification number, first author’s name, publication year, cancer type, patient’s age, type of acupuncture intervention, type of control intervention, the sample size of each intervention group, intervention duration, randomisation, allocation concealment and blinding methods, outcome measures, main outcomes, adverse events, etc. All information was included in a standardised data extraction format. Any disagreements were solved by consensus. A third investigator (DL) made the final judgment when consensus on data extraction could not be obtained through negotiation. If a study was missing information, the corresponding author was contacted (if the contact details were available).

### 2.5 Risk-of-bias assessment

Two reviewers (DL and YO) evaluated the methodological qualities using the RevMan 5.4 software built-in risk bias assessment tool provided by the Cochrane Collaboration (18). The following seven aspects were chiefly included: 1) random sequence generation (selection bias); 2) allocation concealment (selection bias); 3) performance bias: blinded implementation (including participants, investigators, and outcome assessors); 4) detection bias: blinded evaluation of study results; 5) attrition bias: outcome data integrity; 6) reporting bias: selective reporting of results; 7) other biases. All the above biases were assessed and classified as low, unclear, or high risk of bias. Disagreements were discussed between the two reviewers, and if these were unresolved, a third reviewer (DP) participated in the discussion until a consensus was reached.

### 2.6 Evidence quality assessment

The Grading of Recommendations Assessment, Development, and Evaluations (GRADE) approach was used to rate the overall quality of evidence (19). The GRADE guideline has five domains, including the risk of bias, inconsistency, indirectness, imprecision, and potential publication bias. GRADE has four levels of gradings for evidence quality, namely high, moderate, low, and very low. Two researchers (DL and JY) independently performed the assessment, and a third researcher (DP) then reviewed the evaluation. Any disagreement was resolved by discussion with a professional.

### 2.7 Data analysis

Data analysis was performed using RevMan (version 5.4). Continuous variables were assessed using the mean difference (MD) with a 95% confidence interval (CI) when the unit was the same. Else, the standardised MD (SMD) was used. Statistical significance was set at P <0.05. The magnitude of the effect size of the SMD was rated as follows: ≤0.2 indicated a small effect, 0.5 indicated a moderate effect, and ≥0.8 indicated a large effect. The χ^2^ test was used to test for heterogeneity. If I^2^ was <50% in the results, a fixed-effects model was selected to pool the data. Else, a random-effects model was adopted.

If a significant heterogeneity existed between a set of studies, causes of heterogeneity, such as patient characteristics and the degree of variation in the interventions, were explored. The primary outcomes were analysed through subgroup analysis. Sensitivity analysis was performed by excluding each RCT sequentially and comparing the model characteristics to test the robustness of the result. A funnel plot was used to assess the reporting biases if more than 10 trials were included in the meta-analysis. The asymmetry of the funnel plots was evaluated using Egger’s tests, and a P-value of <0.05 represented significant publication bias (20).

## 3 Results

### 3.1 Search results

**Figure 1:**
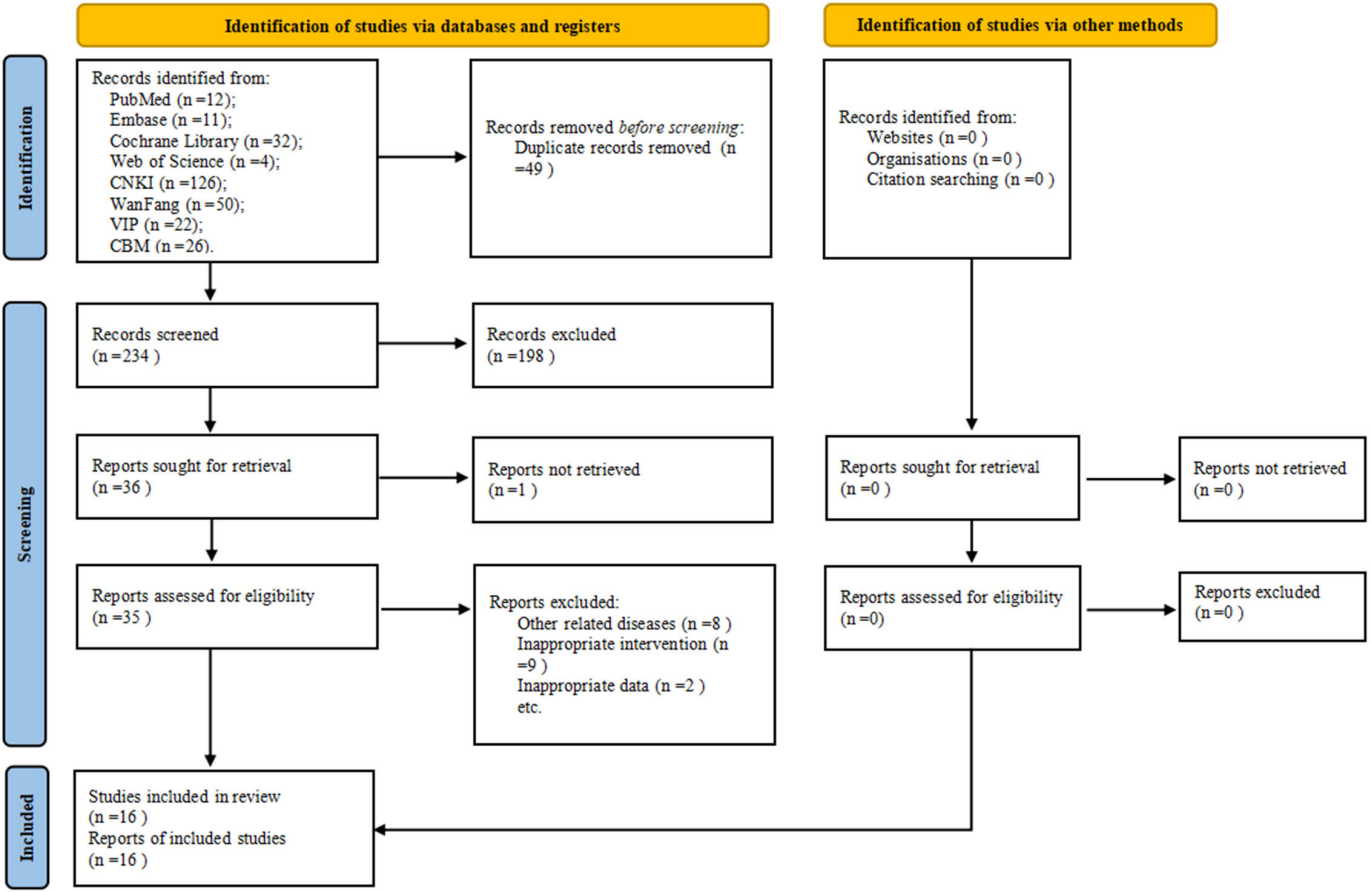
PRISMA flow chart.

A total of 283 potentially relevant articles were identified in the initial database search. After eliminating 49 duplicates, 234 articles were screened, following which 198 articles were eliminated based on the title and abstract screening process. Furthermore, 20 studies were excluded after reviewing full texts based on the eligibility criteria. Eventually, 16 studies (21–36) were included in this meta-analysis (**Figure 1**).

### 3.2 Study characteristics

The included RCTs were published between 2008 and 2022. The main characteristics of the included studies are summarised in **Table 2**.

**Table 2:** Detailed characteristics the included trials.

Study	Age (I/C)		Male (I/C)	Female (I/C)	Intervention group therapy	Control group therapy	Cancer type	Duration and frequency of treatment	Outcomes	Acupuncture points
Ding et al., 2021 (21)	54.23 ±7.98	55.57 ±10.63	18:17	12:13	EA	SA	GC	from postoperative day 1, 20 min, once a day	TFF; TFD; LOS;	GV20 *Baihui*百会, ST36 *Zusanli* 足三里, ST25 *Tianshu* 天枢, PC6 *Neiguan* 内关
Wang et al., 2019 (22)	62.45 ±6.87	63.90 ±6.85	11:9	9:11	EA+RT	RT	RC	from postoperative day 1, 20 min, once a day	TFF; TFD; TBSR	LI9 *Shanglian* 上廉, ST39 *Xiajuxu* 下巨虚, CV12 *Zhongwan* 中脘, ST25 *Tianshu* 天枢
Wang et al., 2022 (23)	66.13 ±15.91	63.63 ±11.64	16:19	14:11	EA+RT	RT	CRC	from postoperative day 1, 30 min, once a day	TFF; TFD; TBSR; LOS	ST36 *Zusanli* 足三里, PC6 *Neiguan* 内关, CV13 *Shangwan* 上脘, CV12 *Zhongwan* 中脘, CV10 *Xiawan* 下脘, ST25 *Tianshu* 天枢, CV6 *Qihai* 气海
Yang et al., 2022 (24)	51.79 ±3.96	52.12 ±4.27	26:28	14:12	EA+RT	RT	GC	from postoperative day 1, 30 min, once a day	TFF; TFD; LOS;	ST36 *Zusanli* 足三里, SP9 *Yinglingqua*阴陵泉, SP6 *Sanyinjiao* 三阴交, ST37 *Shangjuxu* 上巨虚, ST39 *Xiajuxu* 下巨虚
Wu et al., 2020 (25)	62.40 ±11.17	66.00 ±2.12	13:11	2:4	MA+ERAS	ERAS	GC	from postoperative day 1, 30 min, until defecation occurred	TFF; TFD; LOS;	ST37 *Shangjuxu* 上巨虚
Wang et al., 2018 (26)	57.5 ±8.5	58.5 ±8.5	9:10	5:4	NWT+RT	RT	GC	from postoperative day 1, once a day	TFF; TFD; TBSR	ST36 *Zusanli* 足三里, PC6 *Neiguan* 内关, SP6 *Sanyinjiao* 三阴交, LR3 *Taichong* 太冲, KI3 *Taixi* 太溪, LI11 *Quchi*曲池
Xiong 2014 (27)	63.6 ±6.4	64.2 ±5.8	17:16	13:14	PA+RT	RT	OC	from postoperative day 1, 6 h, twice a day	TFF; TFD; TBSR	CV8 *Shenque* 神阙, CV12 *Zhongwan* 中脘
Zhang et al., 2022 (28)	NR	NR	NR	NR	MA+RT	RT	CRC	from postoperative day 4, 30 min, once a day	TFF; TFD; TBSR	CV12 *Zhongwan* 中脘, ST25 *Tianshu* 天枢, PC6 *Neiguan* 内关, SP4 *Gongsun* 公孙, ST36 *Zusanli* 足三里, ST37 *Shangjuxu* 上巨虚
Qian et al., 2017 (29)	59±10	60±11	20:17	10:13	MA+RT	RT	GC	from postoperative day 1, 20 min, once a day	TFF; TBSR; LOS	ST36 *Zusanli* 足三里, ST37 *Shangjuxu* 上巨虚, ST39 *Xiajuxu* 下巨虚
Li et al., 2008 (30)	64	60	10:14	12:9	MA+RT	RT	OC	from postoperative day 1, 30 min, once a day	TFF	ST36 *Zusanli* 足三里
Chen 2021 (31)	34.56 ±8.23	35.50 ±8.83	NR	NR	EA+RT	RT	BGT	from postoperative day 1, 20 min, until defecation occurred	TFF; TFD	LI 10 *Shousanl* 手三里, ST37 *Shangjuxu* 上巨虚, ST36 *Zusanli* 足三里, LI4 *Hegu* 合谷
Lin et al., 2019 (32)	34.57 ±8.29	35.50 ±8.80; 33.53 ±7.39	NR	NR	EA+RT	RT	BGT	from postoperative day 1, 20 min, once a day	TFF; TFD	LI10 *Shousanli* 手三里, TE6 *Zhigou* 支沟, LI4 *Hegu* 合谷, ST36 *Zusanli* 足三里, ST37 *Shangjuxu* 上巨虚, ST39 *Xiajuxu* 下巨虚
Jia 2016 (33)	55.7 ±6.1	56.5 ±6.3; 55.4 ±5.9	16:13:15	14:17 :15	AIT+RT	RT	GC	from postoperative day 1, twice a day	TFF; TFD; TBSR	ST36 *Zusanli* 足三里
Zhong et al., 2016 (34)	57±4.3	55±4.8	17:16	13:14	CEA+RT	RT	RC	NR	TFF; TFD; TBSR; LOS	ST36 *Zusanli* 足三里
Hou et al., 2016 (35)	70±7	70±7	14:10	6:10	TEAS+RT	RT	GC +CRC	from postoperative day 1, 20 min, once a day	TFF	ST36 *Zusanli* 足三里, ST37 *Shangjuxu* 上巨虚
Hsiung et al., 2015 (36)	60.54 ±10.89	64.11 ± 15.60	20:20	6:8	ACUP +RT	RT	GC	from postoperative day 1, 12 min, once a day	TFF; TFD	PC6 *Neiguan* 内关, ST36 *Zusanli* 足三里

I, intervention group; C, control group; NR, not reported; TFF, time to first flatus; TFD, time to first defecation; TBSR, time to bowel sound recovery; LOS, length of hospital stay; EA, electroacupuncture; SA, sham acupuncture; RT, routine treatment; ERAS, enhanced recovery after surgery; MA, manual acupuncture; TEAS, transcutaneous electrical acupoint stimulation; NWT, needle warming therapy; PA, acupoint application; AIT, acupoint injection therapy; CEA, catgut embedding at acupoint; ACUP, acupressure; GC, gastric cancer; CRC, colorectal cancer; OC, oesophageal cancer; BGT, benign gynaecological tumour; GV, governor vessel; ST, stomach; PC, pericardium; LI, large intestine; CV, conception vessel; SP, spleen; LR, liver; KI, kidney; TE, triple energiser.

**Patient characteristics:** The 16 studies (21–36) included 877 participants, of whom 437 belonged to the intervention group, and 440 belonged to the control group. All participants were diagnosed with cancer and had undergone surgery and developed PGD. As for the cancer types, 12 studies (21–26, 28, 29, 33–36) were based on gastrointestinal tumours [including gastric cancer and colorectal cancer), two studies (27, 30) were based on oesophageal cancer, and two studies (31, 32) were based on benign gynaecological tumours.

**Intervention characteristics:** Three types of acupuncture techniques were involved: EA (21–24, 31, 32), MA (25, 28–30), and other acupuncture therapies [including needle warming therapy (26), acupoint application (27), acupoint injection therapy (33), catgut embedding at the acupoint (34), transcutaneous electrical acupoint stimulation (35), and acupressure (36)]. Twenty-one acupoints were used across all studies. The commonly used acupoints were stomach (ST) 25, ST36, ST37, ST39, pericardium (PC) 6, spleen (SP) 6, and conception vessel (CV) 12, which were in the stomach meridian and spleen meridian. In terms of combining acupoints, 11 studies (24–26, 29–36) used a combination of distal acupoints, one study (27) used a combination of proximal acupoints, and four studies (21–23, 28) used a combination of distal–proximal acupoints. Acupuncture was performed after the surgery in all trials. Eleven studies (21, 22, 24, 25, 27–32, 34) reported deqi sensations. Acupuncture retention times in most studies varied from 20 to 30 min. The treatment was administered once a day or twice a day.

**Control characteristics:** RT was administered in 14 RCTs (22–24, 26–36), one study (21) applied SA, and another trial (25) used enhanced recovery after surgery (ERAS).

**Outcomes:** Most studies used TFF, TFD, TBSR, and LOS as subjective or objective outcomes to assess the effect of acupuncture.

### 3.3 Risk of bias

**Figure 2:**
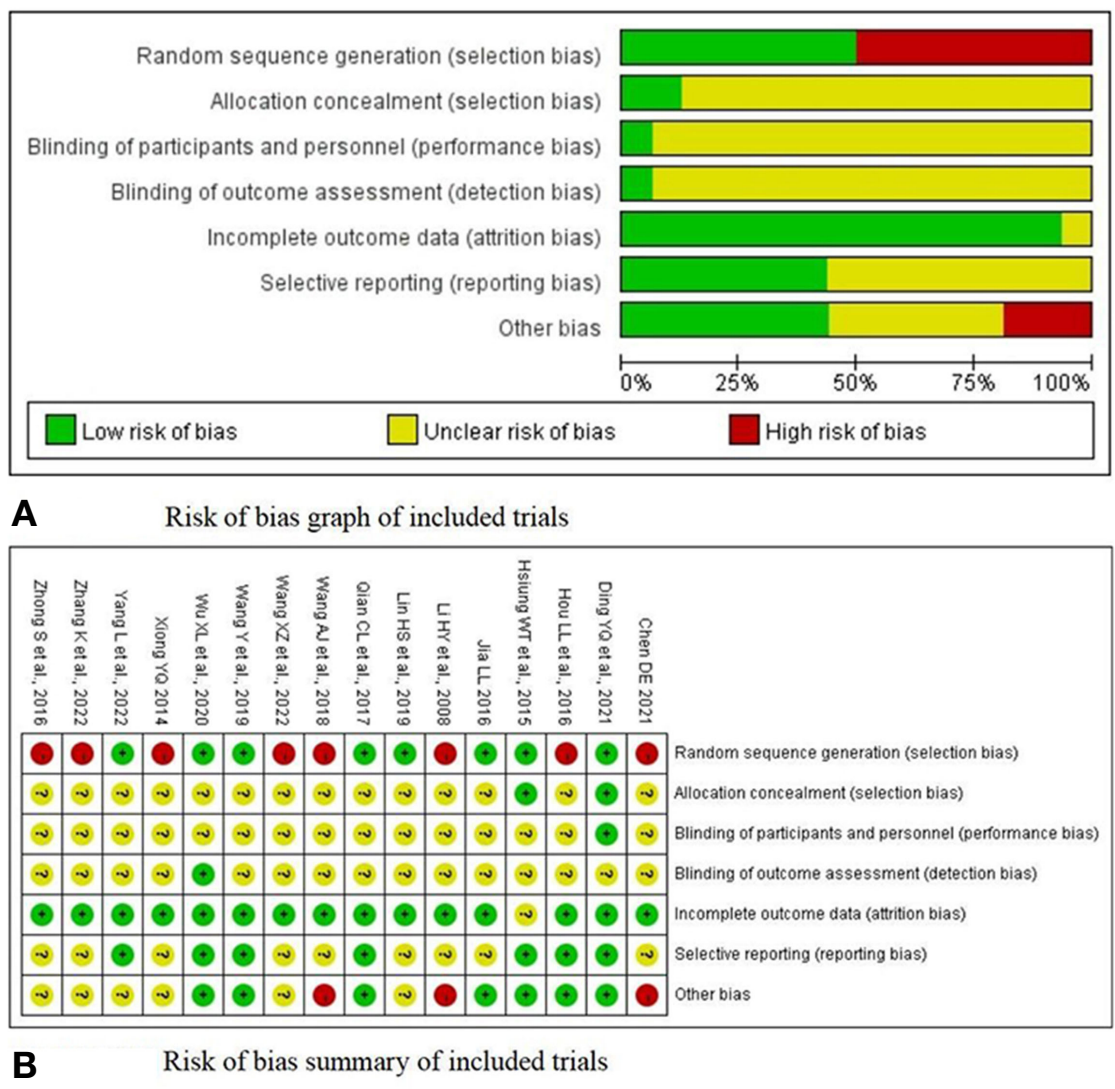
**(A)** Risk of bias graph of included trials. **(B)** Risk of bias summary of included trials.

**Figure 2A** presents the risk of bias in each of the seven domains for all included studies. Regarding the risk of random sequence generation, eight studies (21, 22, 24, 25, 29, 32, 33, 36) explicitly reported the randomisation methods with low bias risks. Only two trials (21, 36) reported regarding allocation concealment *via* the use of sealed envelopes or a computer. Only one study (21) conducted blinding on personnel and participants. In 16 RCTs (21–36), the outcome assessors were not blinded, suggesting a risk of measurement bias. In terms of the outcome data, one study (36), with a loss rate of 10%, was rated as unclear. No dropouts occurred in the 15 other trials (21–35), and the complete outcome indicators were rated as low risk. Seven RCTs (21, 22, 24, 25, 29, 35, 36) were evaluated as having a low risk of bias in the selective reporting domain. Regarding other biases, two studies (26, 30) without specific inclusion and exclusion criteria were assessed as high-risk, and one other study (31) was evaluated as high-risk because it lacked a course of treatment. A summary of the risk of bias in each of the included trials is presented in **Figure 2B**.

### 3.4 Safety

No adverse events were reported in the included studies. Therefore, acupuncture is relatively safe for PGD in cancer.

### 3.5 Meta-analysis

#### 3.5.1 Acupuncture-related therapy vs. RT

**Figure 3:**
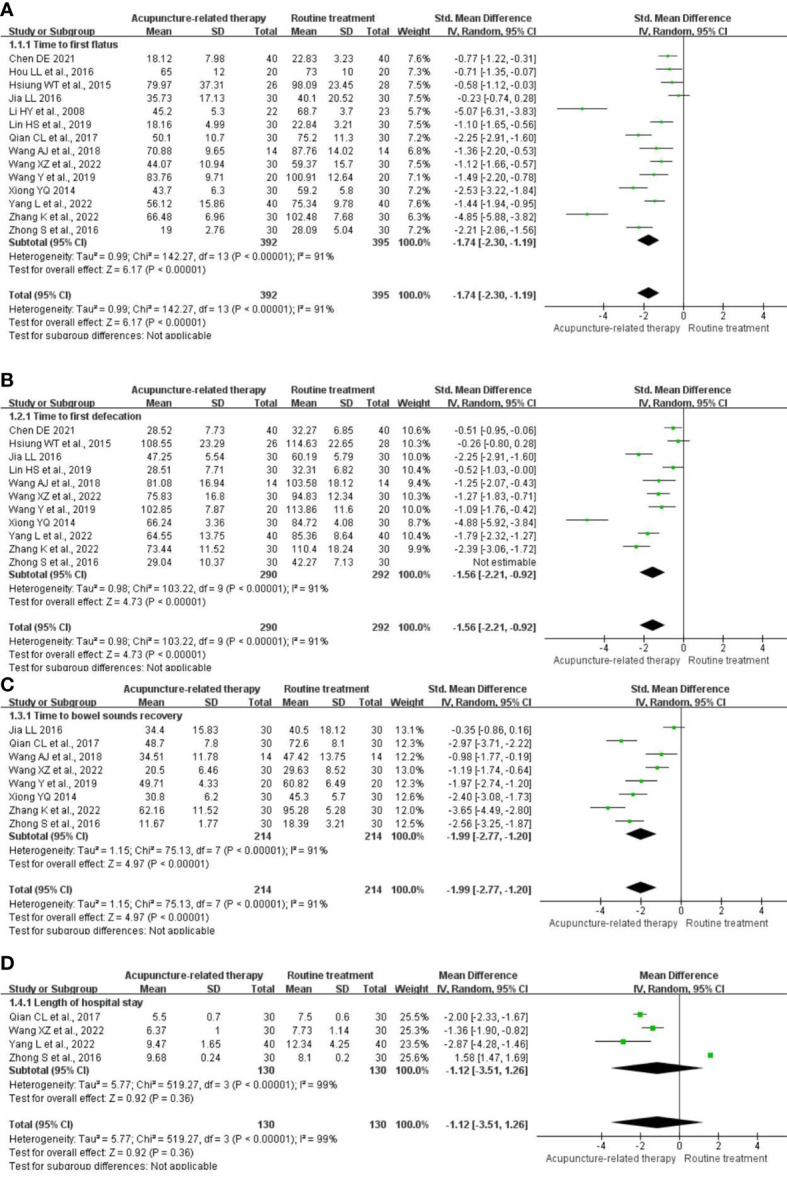
Meta-analysis of acupuncture-related therapy versus routine treatment. **(A)** Meta-analysis of acupuncture-related therapy versus routine treatment for the time to first flatus. **(B)** Meta-analysis of acupuncture-related therapy versus routine treatment for the time to first defecation. **(C)** Meta-analysis of acupuncture-related therapy versus routine treatment for the time to bowel sound recovery. **(D)** Meta-analysis of acupuncture-related therapy versus routine treatment for the length of hospital stay.

**TFF:** Fourteen studies (22–24, 26–36) comprising 787 participants evaluated the change in the TFF. Pooled results indicated that acupuncture-related therapy had a better effect in shortening the TFF compared with RT (SMD = -1.74; 95% CI: -2.30 to -1.19; P <0.00001; I^2^ = 91%; **Figure 3A**).

**TFD:** Eleven trials (22–24, 26–28, 31–34, 36) comprising 582 participants examined the change in the TFD. The analysis revealed that acupuncture-related therapy reduced the TFD compared with RT (SMD = -1.56; 95% CI: -2.21 to -0.92; P <0.00001; I^2^ = 91%; **Figure 3B**).

**TBSR:** Eight RCTs (22, 23, 26–29, 33, 34) comprising 428 participants reported this outcome and revealed significant shortening of the TBSR in the acupuncture-related therapy group compared with the RT group (SMD = -1,99; 95% CI: -2.77 to -1.20; P <0.00001; I^2^ = 91%; **Figure 3C**).

**LOS:** Four studies (23, 24, 29, 34) comprising 260 participants reported the LOS. The acupuncture-related therapy group had a prolonged LOS compared with the RT group (MD = -1.12; 95% CI: -3.51 to 1.26; P = 0.36; I^2^ = 99%; **Figure 3D**).

#### 3.5.2 Acupuncture-related therapy vs. SA

**Figure 4:**
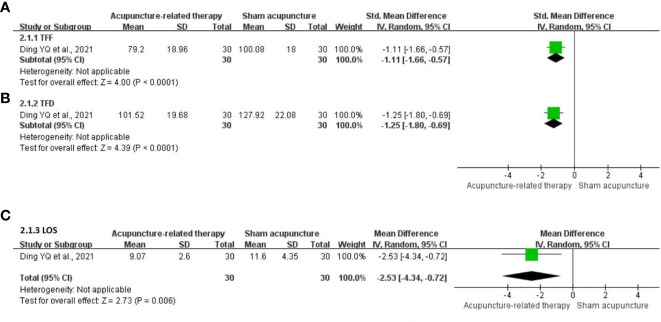
Meta-analysis of acupuncture-related therapy versus sham acupuncture. **(A)** Meta-analysis of acupuncture-related therapy versus sham acupuncture for the time to first flatus. **(B)** Meta-analysis of acupuncture-related therapy versus sham acupuncture for the time to first defecation. **(C)** Meta-analysis of acupuncture-related therapy versus sham acupuncture for the length of hospital stay.

**TFF:** One study (21) comprising 60 participants evaluated the change in the TFF. The pooled results indicated that acupuncture-related therapy had a better effect in shortening the TFF compared with SA (SMD = -1.11; 95% CI: -1.66 to -0.57; P <0.0001; **Figure 4A**).

**TFD:** One trial (21) comprising 60 participants examined the change in the TFD. The analysis revealed that acupuncture-related therapy reduced the TFD compared with SA (SMD = -1.25; 95% CI: -1.80 to -0.69; P <0.0001; **Figure 4B**).

**LOS:** One RCT (21) comprising 60 participants reported the LOS. The LOS was shorter in the acupuncture-related therapy group compared with the SA group (MD = -2.53; 95% CI: -4.34 to -0.72; P = 0.006; **Figure 4C**).

#### 3.5.3 Acupuncture-related therapy vs. ERAS

**Figure 5:**
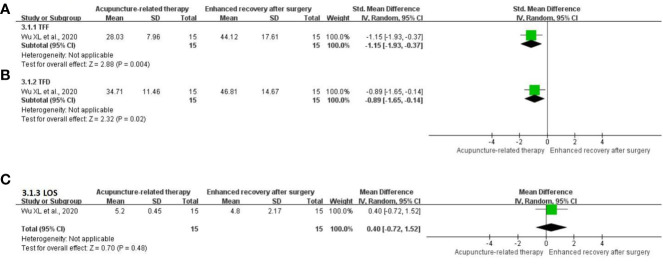
Meta-analysis of acupuncture-related therapy versus enhanced recovery after surgery. **(A)** Meta-analysis of acupuncture-related therapy versus enhanced recovery after surgery for the time to first flatus. **(B)** Meta-analysis of acupuncture-related therapy versus enhanced recovery after surgery for the time to first defecation. **(C)** Meta-analysis of acupuncture-related therapy versus enhanced recovery after surgery for the length of hospital stay.

**TFF:** One study (25) comprising 30 participants evaluated the change in the TFF. The pooled results indicated that acupuncture-related therapy had a better effect in shortening the TFF compared with ERAS (SMD = -1.15; 95% CI: -1.93 to -0.37; P = 0.004; **Figure 5A**).

**TFD:** One trial (25) comprising 30 participants examined the change in the TFD. The analysis revealed that acupuncture-related therapy resulted in a reduction in the TFD compared with ERAS (SMD = -0.89; 95% CI: -1.65 to -0.14; P = 0.02; **Figure 5B**).

**LOS:** One RCT (25) comprising 30 participants reported the LOS. The acupuncture-related therapy group could not shorten the LOS compared with the ERAS group (MD = 0.40; 95% CI: -0.72 to 1.52; P = 0.48; **Figure 5C**).

### 3.6 Sensitivity analysis

No changes were observed in the significant outputs from the meta-analysis by omitting a single study in terms of the TFF, TFD, and TBSR in comparison with RT. These heterogeneities did not influence the stability of the result. However, after excluding the study conducted by Wang et al. (23), Yang et al. (24), and Qian et al. (29), results related to the LOS were not significant, and the heterogeneity significantly increased. However, after eliminating the study by Zhong et al. (34), the results related to the LOS (P <0.00001) revealed significance, and the heterogeneity (I^2^ = 66%) was significantly reduced. A sensitivity analysis was not performed due to the smaller number of studies comparing acupuncture-related treatments with SA and ERAS (**Table 3**).

**Table 3:** Sensitivity analysis.

Comparison	Outcome	Study omitted	Effect Estimate	Test for overall effect P-value	Heterogeneity I^2^ (%)
Routine treatment	TFF	Chen DE 2021	SMD -1.83 [-2.43, -1.23]	<0.00001	91
		Hou LL et al. 2016	SMD -1.83 [-2.42, -1.24]	<0.00001	91
		Hsiung WT et al. 2015	SMD -1.84 [-2.43, -1.25]	<0.00001	91
		Jia LL 2016	SMD -1.86 [-2.43, -1.30]	<0.00001	90
		Li HY et al. 2008	SMD -1.53 [-2.03, -1.03]	<0.00001	89
		Lin HS et al. 2019	SMD -1.80 [-2.41, -1.20]	<0.00001	92
		Qian CL et al. 2017	SMD -1.71 [-2.29, -1.13]	<0.00001	91
		Wang AJ et al. 2018	SMD -1.78 [-2.36, -1.19]	<0.00001	92
		Wang XZ et al. 2022	SMD -1.80 [-2.41, -1.20]	<0.00001	92
		Wang Y et al. 2019	SMD -1.77 [-2.36, -1.18]	<0.00001	92
		Xiong YQ 2014	SMD -1.68 [-2.25, -1.11]	<0.00001	91
		Yang L et al. 2022	SMD -1.78 [-2.39, -1.17]	<0.00001	92
		Zhang K et al. 2022	SMD -1.51 [-1.99, -1.03]	<0.00001	88
		Zhong S et al. 2016	SMD -1.71 [-2.29, -1.13]	<0.00001	91
	TFD	Chen DE 2021	SMD -1.66 [-2.29, -1.04]	<0.00001	90
		Hsiung WT et al. 2015	SMD -1.68 [-2.29, -1.08]	<0.00001	90
		Jia LL 2016	SMD -1.48 [-2.09, -0.87]	<0.00001	90
		Lin HS et al. 2019	SMD -1.66 [-2.29, -1.03]	<0.00001	90
		Wang AJ et al. 2018	SMD -1.58 [-2.21, -0.95]	<0.00001	91
		Wang XZ et al. 2022	SMD -1.58 [-2.24, -0.93]	<0.00001	91
		Wang Y et al. 2019	SMD -1.60 [-2.24, -0.96]	<0.00001	91
		Xiong YQ 2014	SMD -1.26 [-1.72, -0.81]	<0.00001	84
		Yang L et al. 2022	SMD -1.53 [-2.17, -0.89]	<0.00001	91
		Zhang K et al. 2022	SMD -1.46 [-2.07, -0.86]	<0.00001	90
		Zhong S et al. 2016	SMD -1.56 [-2.21, -0.92]	<0.00001	91
	TBSR	Jia LL 2016	SMD -2.23 [-2.92, -1.55]	<0.00001	85
		Qian CL et al. 2017	SMD -1.85 [-2.68, -1.03]	< 0.0001	91
		Wang AJ et al. 2018	SMD -2.13 [-3.00, -1.27]	<0.00001	92
		Wang XZ et al. 2022	SMD -2.11 [-3.02, -1.20]	<0.00001	91
		Wang Y et al. 2019	SMD -1.99 [-2.88, -1.10]	<0.0001	92
		Xiong YQ 2014	SMD -1.93 [-2.81, -1.05]	<0.0001	92
		Zhang K et al. 2022	SMD -1.76 [-2.51, -1.01]	<0.00001	89
		Zhong S et al. 2016	SMD -1.91 [-2.78, -1.04]	<0.0001	91
	LOS	Qian CL et al. 2017	MD -0.82 [-3.38, 1.75]	0.53	99
		Wang XZ et al. 2022	MD -1.05 [-3.98, 1.88]	0.48	100
		Yang L et al. 2022	MD -0.59 [-3.29, 2.11]	0.67	100
		Zhong S et al. 2016	MD -1.88 [-2.49, -1.26]	<0.00001	66

SMD, standard mean difference; MD, mean difference; TFF, time to first flatus; TFD, time to first defecation; TBSR, time to bowel sounds recovery; LOS, length of hospital stay.

### 3.7 Subgroup analysis

Due to the limited number of studies, only the primary outcomes, TFF and TFD, were analysed. The subgroups were based on the following characteristics: 1) acupuncture techniques: EA, MA, or other acupuncture therapies; 2) cancer types: gastrointestinal tumours (including gastric cancer and colorectal cancer), oesophageal cancer, or benign gynaecological tumours; 3) acupoint combinations: a combination of distal acupoints, a combination of proximal acupoints, or distal–proximal acupoint combination. The results are presented in **Table 4**.

**Table 4:** Subgroup analysis.

Outcome	Subgroup		Studies	Patients	Effect Sizes SMD	95%CI	Heterogeneity I^2^	P value
TFF	Acupuncture technique	Electroacupuncture	6	380	-1.13	[-1.35,-0.91]	0%	<0.00001
		Manual acupuncture	4	195	-3.28	[-5.05,-1.51]	94%	0.0003
		Other acupuncture therapies	6	302	-1.25	[-2.02,-0.49]	89%	0.001
	Cancer type	Gastroenteric tumors	12	632	-1.49	[-2.01,-0.97]	88%	<0.00001
		Oesophageal cancer	2	105	-3.74	[-6.24,-1.25]	92%	0.003
		Benign gynaecological tumours	2	140	-0.90	[-1.25,-0.55]	0%	<0.00001
	Acupoints combination	Combination of distal acupoints	11	597	-1.43	[-1.96,-0.90]	88%	<0.00001
		Combination of proximal acupoints	1	60	-2.53	[-3.22,-1.84]	-	<0.00001
		Distal-proximal acupoints combination	4	220	-2.07	[-3.36,-0.79]	93%	0.002
TFD	Acupuncture technique	Electroacupuncture	6	380	-1.06	[-1.48,-0.64]	73%	<0.00001
		Manual acupuncture	2	90	-1.65	[-3.12,-0.19]	88%	0.03
		Other acupuncture therapies	5	262	-1.97	[-3.23,-0.72]	94%	0.002
	Cancer type	Gastroenteric tumors	10	532	-1.39	[-1.79,-0.99]	76%	<0.00001
		Oesophageal cancer	1	60	-4.88	[-5.92,-3.84]	-	<0.00001
		Benign gynaecological tumours	2	140	-0.51	[-0.85,-0.17]	0%	0.003
	Acupoints combination	Combination of distal acupoints	8	452	-1.10	[-1.60,-0.60]	83%	<0.0001
		Combination of proximal acupoints	1	60	-4.88	[-5.92,-3.84]	–	<0.00001
		Distal-proximal acupoints combination	4	220	-1.48	[-2.03,-0.94]	69%	<0.00001

TFF, time to first flatus; TFD, time to first defecation; SMD, standardized mean difference; 95%CI, 95% confidence interval.

The subgroup analysis revealed that studies comprising all acupuncture techniques, particularly EA, had a significant effect on reducing the TFF and TFD ([TFF: SMD = -1.13;95% CI: -1.35 to -0.91; P <0.00001; I^2^ = 0%]; [TFD: SMD = -1.06; 95% CI: -1.48 to -0.64; P <0.00001; I^2^ = 73%]). Regarding the cancer type, acupuncture had a significant effect on improving the TFF and TFD in all cancer types included in this review; in particular, it had a more positive effect on improving the TFF and TFD in benign gynaecological tumours ([TFF: SMD = -0.90; 95% CI: -1.25 to -0.55; P <0.00001; I^2^ = 0%]; [TFD: SMD = -0.51; 95% CI: -0.85 to -0.17; P = 0.003; I^2^ = 0%]). In the analysis based on the combination of acupoints, studies that applied a combination of distal acupoints showed a positive effect on reducing the TFF (SMD = -1.43; 95% CI: -1.96 to -0.90; P <0.00001; I^2^ = 88%), and the study applying a combination of distal–proximal acupoints had a significant improvement in reducing the TFD (SMD = -1.48; 95% CI: -2.03 to -0.94; P <0.00001; I^2^ = 69%).

### 3.8 Publication bias

**Figure 6:**
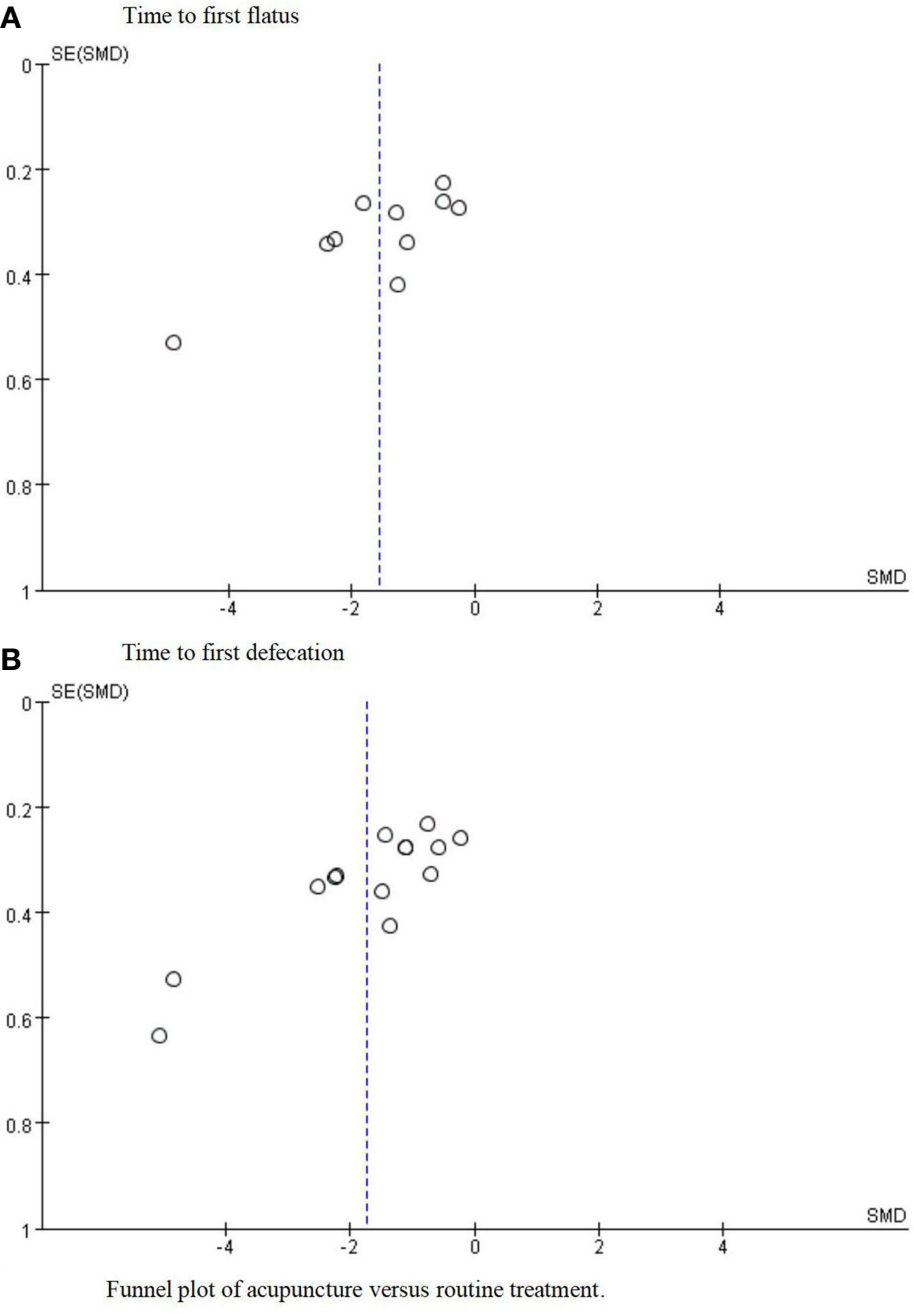
Funnel plot of acupuncture versus routine treatment. **(A)** Funnel plot of acupuncture versus routine treatment for the time to first flatus. **(B)** Funnel plot of acupuncture versus routine treatment for the time to first defecation.

**Figure 7:**
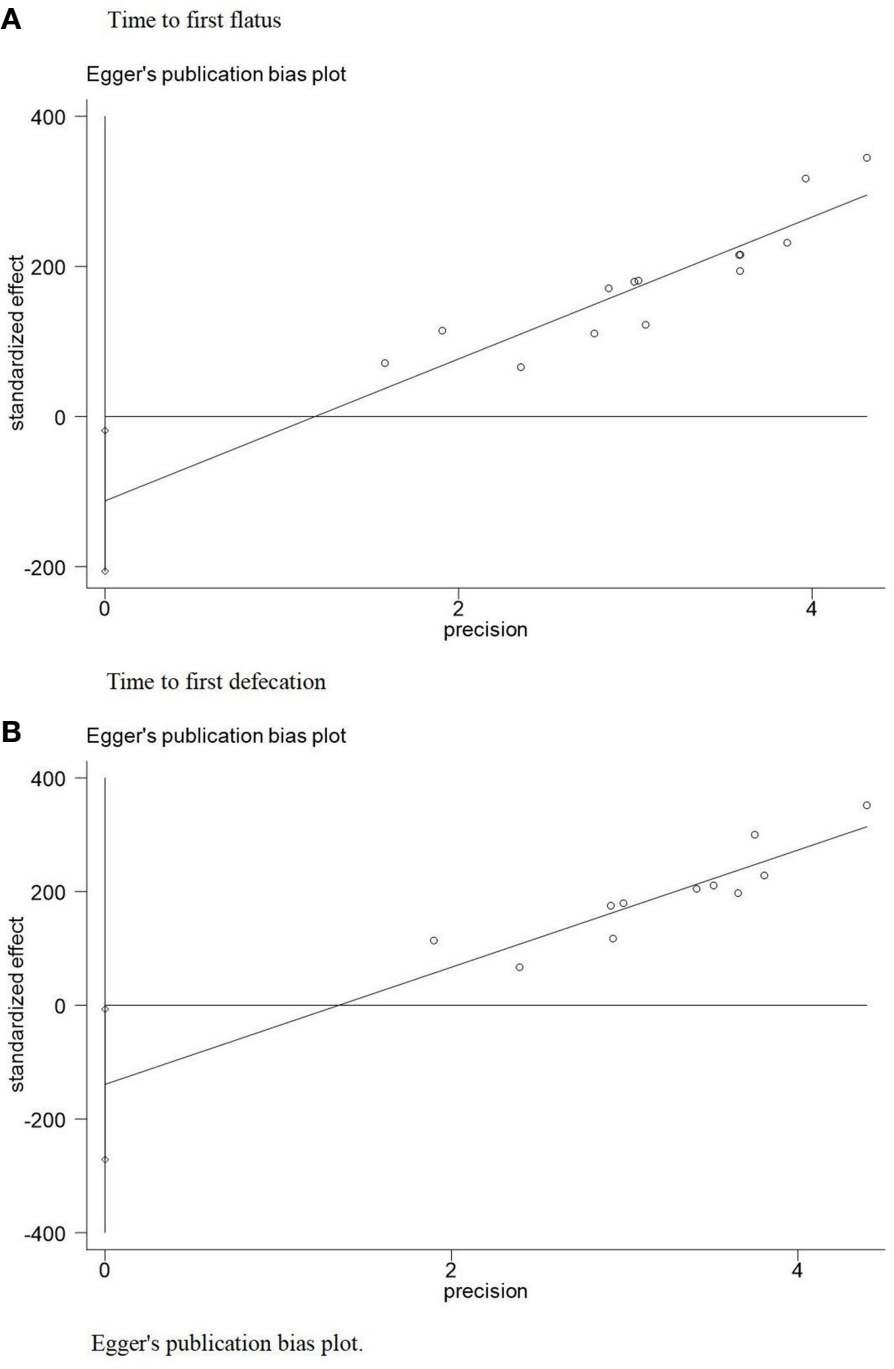
Eager’s publication bias plot. **(A)** Egger's publication bias plot for the time to first flatus. **(B)** Egger's publication bias plot for the time to first defecation.

The funnel plot of 14 trials (22–24, 26–36) included in the meta-analysis for TFF (**Figure 6A**) revealed approximate symmetry between them. Additionally, the funnel plot of 11 trials (22–24, 26–28, 31–34, 36) that reported the TFD (**Figure 6B**) revealed a similar tendency. Egger’s test demonstrated no obvious publication bias (TFF: P = 0.023; TFD: P = 0.041) (**Figure 7**).

### 3.9 Quality of evidence

**Figure 8:**
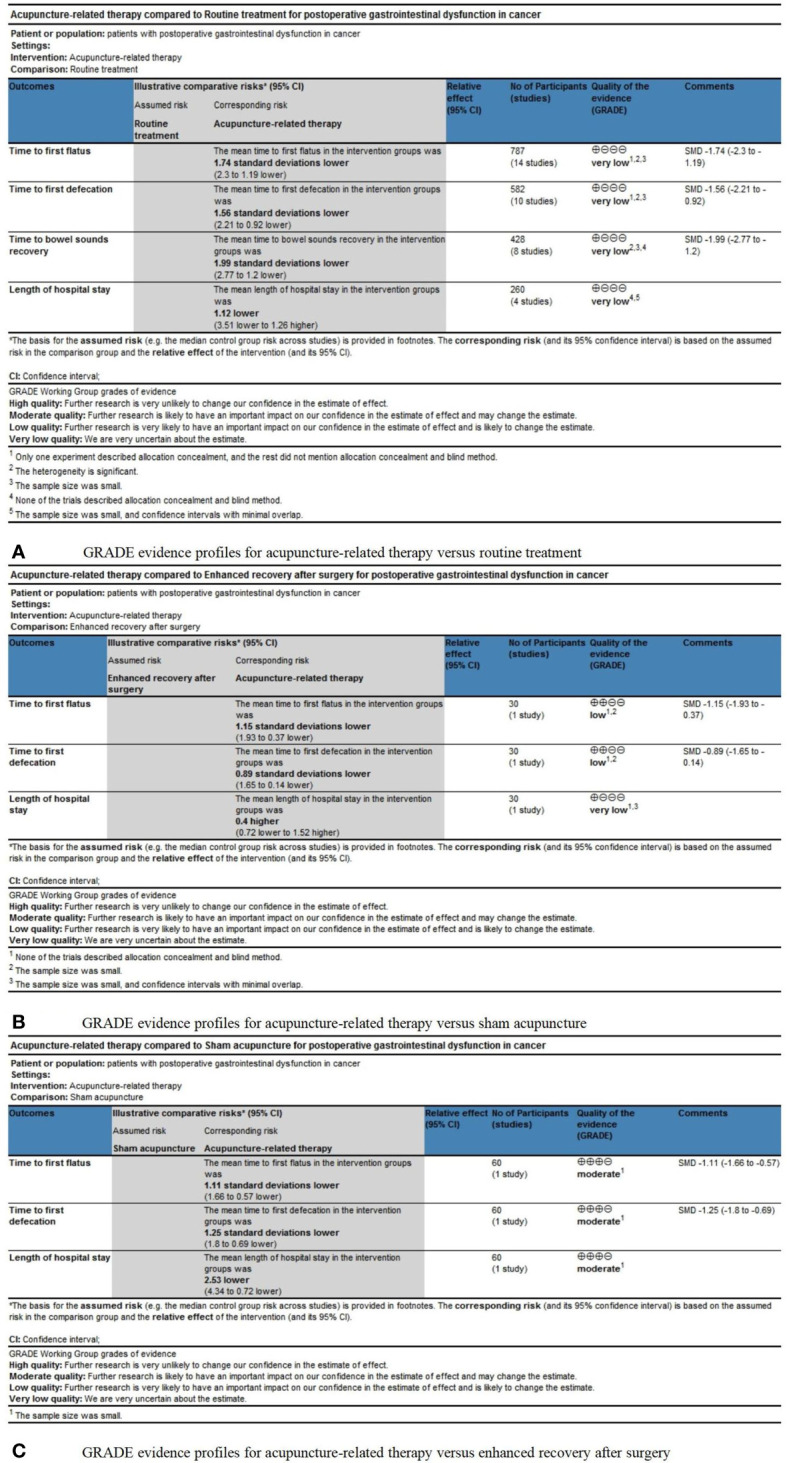
**(A)** GRADE evidence profiles for acupuncture-related therapy versus routine treatment. **(B)** GRADE evidence profiles for acupuncture-related therapy versus sham acupuncture. **(C)** GRADE evidence profiles for acupuncture-related therapy versus enhanced recovery after surgery.

A systematic review of the results was performed using the Cochrane Collaboration Network GRADE approach (**Figure 8**). The systematic analysis comprised four outcomes in the acupuncture-related therapy group compared with the RT group (**Figure 8A**), three outcomes in the acupuncture-related therapy group compared with the SA group (**Figure 8B**), and three outcomes in the acupuncture-related therapy group compared with the ERAS group (**Figure 8C**). TFF and TFD were the key outcomes, while TBSR and LOS were the secondary outcomes. The GRADE profile indicated that the quality of evidence was low for most of the outcomes, mainly due to the risk of bias, unexplained inconsistencies, and the small sample sizes.

## 4 Discussion

### 4.1 Summary of the main findings

This systematic review and meta-analysis aimed to assess the effectiveness and safety of acupuncture for PGD in cancer, including 16 RCTs with 877 participants.

Low to moderate quality of evidence suggested that acupuncture could effectively reduce the TFF, TFD, and TBSR compared with RT, SA, and ERAS; however, acupuncture did not shorten the LOS compared with RT and ERAS. The subgroup analysis revealed that all acupuncture techniques, particularly EA, could significantly reduce the TFF and TFD. Acupuncture effectively reduced the TFF and TFD in all cancer types included in this review, particularly in benign gynaecological tumours. Besides, acupuncture treatment with a combination of distal acupoints could reduce the TFF and TFD, and the combination of distal–proximal acupoints could significantly reduce the TFD. The sensitivity analysis and publication bias supported the stability of the overall results. No adverse events were reported in any of the trials. Overall, acupuncture is relatively safe for PGD in cancer.

### 4.2 Possible explanations for the present findings

In the literature on TCM, PGD can be classified into the categories of constipation, ruffian, abdominal pain, vomiting, etc. TCM believes that surgical trauma easily causes blood deficiency, thereby causing postoperative qi and blood damage in patients. On the other hand, intraoperative bleeding, postoperative fasting, and prolonged lying and rest lead to qi and blood deficiency (37). Qi deficiency is unfavourable for blood flow, blood deficiency easily affects qi function, and this vicious cycle leads to gastrointestinal dysfunction (24).

The mechanisms causing PGD mainly comprise inflammatory reactions caused by surgical trauma and stimulation, side effects of anaesthesia and analgesic drugs, nervous system disorders, dysregulation of gastrointestinal hormonal activity, etc (38, 39). Gastrointestinal hormones regulate gastrointestinal motor function in the form of excitation or inhibition through endocrine secretion, lumen secretion, and neurotransmitters (40). Surgical trauma can affect gastrointestinal function by inhibiting gastrointestinal hormonal secretion, directly resulting in postoperative gastrointestinal diseases (41).

Clinical and experimental studies have reported that postoperative acupuncture can promote gastrointestinal hormonal secretion, enhance the gastrointestinal peristaltic wave, promote the recovery of postoperative gastrointestinal function, and reduce postoperative complications (42–48). Through the parasympathetic efferent pathway, acupuncture can directly induce motility acceleration to restore gastrointestinal transport (49, 50). The studies by Yang et al. (51, 52) revealed that EA could alleviate intestinal inflammation by activating the α7 nicotinic acetylcholine receptor-mediated Janus kinase 2/signal transducer and activator of transcription 3 signalling pathway. Deng et al. (53, 54) reported that acupuncture could improve postoperative gastrointestinal motility by facilitating the recovery of the interstitial cells of Cajal. In addition, EA can trigger nerve excitability conduction and cause regular muscle diastotor contraction, thereby improving the blood circulation at the lesion site, regulating vasomotor function, eliminating nerve oedema, and promoting the recovery of nerve and muscle function (55). Studies have reported that EA can effectively shorten the TBSR, accelerate postoperative exhaust and defecation, and promote gastrointestinal functional recovery (56, 57).

Combining acupoints under the guidance of TCM is one of the significant factors affecting the efficacy of acupuncture. Combining acupoints comprises a combination of distal acupoints, a combination of proximal acupoints, or a combination of distal–proximal acupoints (58). Distal acupoints refer to the points away from the gastrointestinal area, and proximal acupoints refer to the points close to the gastrointestinal area. Evidence from this review revealed that among the combination of acupoints in the included studies, ST25, ST36, ST37, ST39, PC6, SP6, and CV12 were the commonly used acupoints. Among them, ST36 was the most frequently used acupoint, along with other distal acupoints, such as ST37, ST39, SP6, and PC6. The most distal acupoints might be chosen to avoid infection caused by contact with the surgical incision. Modern studies have reported that acupuncture for ST36 could improve gastrointestinal smooth muscle excitability, accelerate gastrointestinal peristalsis, and promote exhaust and defecation (59), and EA for ST36 could promote the secretion of endogenous β-endorphin and inhibit the release of 5-hydroxytryptamine and prostaglandin E2, thus producing good postoperative analgesia (60). Reportedly, EA for ST36 can activate the vagal pathway in the dorsal solitary bundle nucleus-vagal motor nucleus, thus enhancing gastrointestinal activity (61).

### 4.3 Comparison with other reviews and strengths of this review

A previous systematic review and meta-analysis (62) evaluated the effect of acupuncture for treating postoperative ileus in colorectal cancer and revealed that acupuncture could help bring about postoperative ileus recovery in colorectal cancer. Three systematic reviews and meta-analyses (63–65) evaluated the effects of acupuncture for PGD in patients with gastric cancer, and the results revealed that acupuncture promoted PGD recovery in gastric cancer. However, understanding the concept of PGD was relatively vague; therefore, there were controversies regarding patient selection, interventions, and outcome criteria, resulting in the inclusion of inaccurate RCTs.

According to this review, PGD varies in definition, symptoms, and disease severity relative to postoperative ileus; therefore, studies on cancer postoperative ileus was not included in this review. Furthermore, for precise interventions, acupuncture combined with other TCM-related therapies was not included in this study. Most importantly, previous systematic reviews and meta-analyses focused on acupuncture for PGD in gastric cancer and colorectal cancer. However, this review does not limit the cancer type, anticipating that acupuncture could help rehabilitate patients with PGD in cancer and provide evidence for acupuncture interventions for PGD in cancer.

### 4.4 Limitations of included studies

#### 4.4.1 Evidence and methodological quality

The GRADE profile revealed a low quality of evidence for most results, mainly due to methodological limitations such as randomness, risk of bias, unexplained inconsistencies, and the sample sizes of the included studies. Among the 16 enrolled RCTs, eight studies (23, 26–28, 30, 31, 34, 35) did not provide a detailed description of the randomisation process. In addition, only two trials (21, 36) provided information on allocation concealment. Only one RCT (21) blinded the participants and personnel, and another trial (25) blinded the outcome assessment. One study (36), with a loss rate of 10%, was rated as having incomplete outcome data. Furthermore, nine studies (23, 26–28, 30–34) did not provide details of whether there was a selective reporting bias. Moreover, three RCTs (26, 30, 31) did not provide the baseline data and exclusion criteria. These various bias types might have contributed to the extremely low methodological quality.

#### 4.4.2 Inconsistent interventions

The fewer number of included studies was responsible for the limitations of the interventions. Particularly in the control group intervention, there existed where only one RCT was included, such as acupuncture-related therapy vs. SA (21) and acupuncture-related therapy vs. ERAS (25). In addition, the included RCTs also varied in acupoint selection, starting intervention time, the number of sessions, frequency, time for needle retention, and acupuncture depth, all of which might have contributed to bias.

#### 4.4.3 Other limitations

First, in the included studies, most of the patients were from China; therefore, our evidence should be used cautiously in other countries. Second, the meta-analysis results revealed significant heterogeneity; however, it was not well resolved by sensitivity and subgroup analyses. The methodological quality evaluation and factors that might cause PGD, such as preoperative anxiety (37, 66, 67), anaesthesia and analgesia methods (68–71), intraoperative operation (72, 73), internal environment disorder (74, 75), might explain the source of heterogeneity. Despite no adverse events being reported in the included trials, the safety of acupuncture could not be adequately evaluated due to the limited number of included RCTs. Finally, the included studies had no health economic data or relevant health economic analyses.

### 4.5 Outlook

On analysing the studies included, we observed that many RCTs lacked attention to the follow-up of acupuncture for PGD in cancer. We anticipate that there will be more high-quality trials involving more acupuncture techniques and cancer types, focusing on the acupuncture parameters, such as frequency, stimulation, duration, and combination of acupoints, to formulate an optimal acupuncture treatment plan for PGD in cancer, for further determining the effectiveness and safety of acupuncture in populations beyond China. Meanwhile, the evidence in this review indicated that acupuncture does not shorten the LOS, and we anticipate future studies to evaluate the health economics of acupuncture for PGD in cancer by shortening the LOS to provide patients with more effective and affordable green therapy.

## 5 Conclusion

Acupuncture is effective and relatively safe for treating PGD in cancer. Acupuncture can effectively improve the TFF, TFD, and TBSR in patients with PGD for gastroenteric tumours (including gastric cancer and colorectal cancer), oesophageal cancer, and benign gynaecological tumours. However, the existing evidence does not imply that acupuncture can shorten the LOS in patients with PGD in cancer. Nevertheless, the methodological quality of the included studies was generally poor, and further well-designed, high-quality, large-scale, multicentre studies involving more acupuncture techniques, cancer types, and acupoint combinations for PGD in cancer are warranted to verify our findings.

## Data availability statement

The original contributions presented in the study are included in the article/supplementary material. Further inquiries can be directed to the corresponding author.

## Author contributions

DL, YO, and DP conceived and designed this study. YO and KW searched the databases. DL, LL, and QZ participated in the study selection and data extraction. DL, YO, LL, QZ, and JY interpreted and assessed the data. DL and YO depicted the tables and figures. DL, YO, LL, KK and KW drafted the manuscript. DP revised the manuscript. All authors approved the submitted version of the manuscript.
